# Challenges in co-designing an intervention to increase mobility in older patients: a qualitative study

**DOI:** 10.1108/JHOM-02-2020-0049

**Published:** 2021-04-09

**Authors:** Jeanette Kirk, Thomas Bandholm, Ove Andersen, Rasmus Skov Husted, Tine Tjørnhøj-Thomsen, Per Nilsen, Mette Merete Pedersen

**Affiliations:** Department of Clinical Research and Department of Public Health, Nursing, Aarhus University , Aarhus, Denmark; Amager and Hvidovre Hospital, University Hospital of Copenhagen , Hvidovre, Denmark; Department of Clinical Research, Copenhagen University Hospital, Amager and Hvidovre , Hvidovre, Denmark; Department of Orthopedic Surgery, Copenhagen University Hospital, Amager and Hvidovre , Hvidovre, Denmark; Department of Physical and Occupational Therapy, Physical Medicine and Rehabilitation Research-Copenhagen (PMR-C), Copenhagen University Hospital, Amager and Hvidovre , Hvidovre, Denmark; Department of Clinical Research, Amager and Hvidovre Hospital, University of Copenhagen , Hvidovre, Denmark; Department of Health and Social Context, National Institute of Public Health, University of Southern Denmark , Sønderborg, Denmark; Department of Health, Medical and Caring Sciences, Linköping University , Linköping, Sweden

**Keywords:** Co-design, Qualitative, User-engagement, Mobility, Older medical patients

## Abstract

**Purpose:**

The aim of this study is to explore and discuss key challenges associated with having stakeholders take part in co-designing a health care intervention to increase mobility in older medical patients admitted to two medical departments at two hospitals in Denmark.

**Design/methodology/approach:**

The study used a qualitative design to investigate the challenges of co-designing an intervention in five workshops involving health professionals, patients and relatives. “Challenges” are understood as “situations of being faced with something that needs great mental or physical effort in order to be done successfully and therefore tests a person's ability” (Cambridge Dictionary). Thematic content analysis was conducted with a background in the analytical question: “What key challenges arise in the material in relation to the co-design process?”.

**Findings:**

Two key challenges were identified: engagement and facilitation. These consisted of five sub-themes: recruiting patients and relatives, involving physicians, adjusting to a new researcher role, utilizing contextual knowledge and handling ethical dilemmas.

**Research limitations/implications:**

The population of patients and relatives participating in the workshops was small, which likely affected the co-design process.

**Practical implications:**

Researchers who want to use co-design must be prepared for the extra time required and the need for skills concerning engagement, communication, facilitation, negotiation and resolution of conflict. Time is also required for ethical discussions and considerations concerning different types of knowledge creation.

**Originality/value:**

Engaging stakeholders in co-design processes is increasingly encouraged. This study documents the key challenges in such processes and reports practical implications.

## 1. Introduction

Extensive research in the field of implementation science has demonstrated the difficulties of disseminating, implementing and scaling up interventions despite their efficacy and effectiveness ( Balas and Boren, 2000 ; Lane-Fall *et al.* , 2019 ). Recognition of these challenges has led to calls for intervention designs that are more clinically relevant and yields more implementable interventions ( Reis *et al.* , 2016 ), for example, hybrid effectiveness designs ( Curran *et al.* , 2012 ) and participatory designs ( Jull *et al.* , 2017 ; Mercer *et al.* , 2007 ), which acknowledge stakeholder engagement and emphasize implementation goals alongside intervention outcomes ( Proctor *et al.* , 2011 ).

Increasing recognition of the importance of engaging stakeholders in research had led to extensive but sometimes conflicting literature focusing on understanding experiences of engagement ( Fransman, 2018 ). Engagement may simply involve telling people about research ( Caress *et al.* , 2012 ), which is ethically required; other times, engagement means carrying out research together with stakeholders as active partners and where cooperation is visibly required. Engaging stakeholders manifests itself through methods such as co-design and co-creation, which utilize user-centred and participatory design techniques to develop more person-centred public services and implement improvements and innovations ( Voorberg *et al.* , 2015 ). Co-design and co-creation are based on the principles of co-production ( Durose and Richardson, 2016 ), which is understood as a process by which “citizens can play an active role in producing public goods and services of consequence to them” ( Ostrom, 1996 ).

Health care policies and practice have increasingly been oriented towards engaging patients and using co-design methods to involve patients and relatives in quality improvement and research. However, despite the “engagement movement”, patients are still often given a passive role with staff making the most important decisions ( Robert *et al.* , 2015 ). In this article, we use the term engagement to reflect an interest in stakeholders (especially patients) as partners in research ( Locock and Boaz, 2019 ).

Even though collaborative research practices such as co-design are well established within health care quality improvement research and practice, co-design is relatively new in biomedical research ( Locock and Boaz, 2019 ). Biomedical research is the broad area of science that looks for ways to prevent and treat diseases that cause illness and death in people and in animals. This study is focused around an intervention to prevent low mobility among older medical hospitalized patients. Low mobility increases the risk of functional decline, loss of independence and death ( Brown *et al.* , 2004 ; Zisberg *et al.* , 2011 ). In the scholarly field of co-design research, there are ongoing debates about how co-design is applied, interpreted and evaluated ( Robert *et al.* , 2015 ). Also, there is a lack of rigorous evaluation of the effectiveness and cost-effectiveness of co-designed interventions and policies ( Clarke *et al.* , 2017 ; Durose *et al.* , 2017 ). These debates about the applicability of co-design have spread into biomedical research, particularly when designing interventions to be tested in researcher-controlled randomized controlled trials that are more tightly structured and regulated than anything in the field in which co-design was developed and thus raise questions about the transferability of co-design practices. Our study case focuses on co-designing a mobility intervention, which has to be tested in a randomized controlled trial involving researchers, health professionals, patients and relatives; our aim was to explore and discuss the key challenges associated with having stakeholders take part in the design of a health care intervention. Stakeholders are defined as “individuals, organizations or communities that have a direct interest in the process and outcomes of a project, research or policy endeavour” ( Deverka *et al.* , 2012 ).

## 2. Theory

### 2.1 The engagement movement

Historically, the engagement movement has influenced many fields, including the design research field where designers have moved increasingly closer to the users in the entire design process and of what and when to design ( Sanders and Stappers, 2008 ). The movement is operationalized through two approaches. The user-centred approach is a US-driven phenomenon, where the designer designs for the users, and the users are viewed as passive subjects. The participatory approach is a Northern European phenomenon, where the users are viewed as active subjects and thereby as partners and where the designer designs together with the users ( Sanders, 2006 ). The notion of co-production and co-design has emerged from the participatory approach, in which co-production originally referred to the joint working of people, who are not in the same organization, to produce goods or services ( Durose *et al.* , 2017 ; Ostrom, 1996 ). The engagement movement in health care is characterized by a process of building the capacity of patients, families and health care providers to facilitate and support the active involvement of patients in their own care. The goal of this process is to enhance safety, quality and people-centredness of health care service delivery ( Valderas, 2016 ). Today, co-production and co-design are mainstream terms used in health research ( Locock and Boaz, 2019 ) and are widely seen in current policy agendas as the next logical step to patient and public engagement and as a way of incorporating people's expertise into health and social services and research ethics in more substantive and meaningful ways. The engagement movement is visible in different co-processes in health care: (1) co-commissioning of services, which includes co-planning of health and social policy, co-prioritization of services and co-financing of services; (2) co-design of services; (3) co-delivery of services, which includes co-managing and co-performing services and (4) co-assessment, which includes co-monitoring and co-evaluating services ( Batalden *et al.* , 2016 ). Co-design in this article refers to the creativity of designers and people, who are not trained in design, working together in the design development process ( Sanders and Stappers, 2008 ). This definition reflects a shift of focus from products to broader human goals and propagates the ability of co-design to tackle complex societal problems.

### 2.2 Change in participant roles

Co-design has an impact on the roles of the stakeholders in the design or research process where the stakeholder is given the position of “expert of his experience” and thereby plays a larger role in knowledge development, idea generation and concept development ( Sanders and Stappers, 2014a , b ). For the stakeholders to take on this position, they must be given appropriate tools to express themselves, for example, pictures or clay ( Leask *et al.* , 2019 ; Sanders *et al.* , 2010 ; Sanders and Stappers, 2014a , b ). A key dimension of co-design is the creative act of making products, which is referred to as the concept of making (henceforth: making) and includes using probes, generative toolkits and testing protypes, often in iterative cycles ( Sanders and Stappers, 2014a , b ). The engagement movement has not only led to change in roles but also in activities. Previously, only designers used making to shape the future. Because of the engagement movement, we also see non-designers working together with, for example, researchers using making as a way to make sense of the future. In this project, the concept of making is operationalized by making an intervention that engages health professionals, patients and relatives. In co-design, the concept of making cannot be separated from activities such as telling and enacting ( Sanders and Stappers, 2014a , b ). Co-design processes are often organized in workshops. However, discussions in the research design field have considered whether the format of workshops, rather than the format of a laboratory, provides a more relevant framework for understanding the activities in co-design processes where stakeholders are expected to collaboratively explore possibilities in a transparent, systematic and scalable process ( Binder *et al.* , 2011 ; Binder and Brandt, 2008 ). Also, the role of the researcher has changed as a ripple effect of the engagement movement. Previously, the researcher served as a translator between the designer and the stakeholders, but in contemporary co-design, the researcher assumes the role of a facilitator to support stakeholders' expressions of creativity. This involves leading, guiding and providing scaffolds (supporting the stakeholders) as well as clean slates to encourage stakeholder creativity ( Bombard *et al.* , 2018 ; Sanders and Stappers, 2008 ).

### 2.3 Advantages and disadvantages of co-design

Advantages of using co-design have been described, with reference to substantive, instrumental, normative and political arguments ( Oliver *et al.* , 2019 ). A substantive argument is where stakeholder engagement is undertaken to improve the quality of the research, including improved credibility of the results ( Barber *et al.* , 2011 ; Stewart and Liabo, 2012 ). Increasingly, research institutions such as universities and funders have recognized that stakeholder engagement in the research process can improve the impact of research in the wider society ( Fransman, 2018 ). Despite this, involvement of stakeholders is a topic of ongoing discussion in research institutions ( Oliver *et al.* , 2019 ). Instrumental arguments for stakeholder engagement are based on a desire to utilize research findings in an effective way, including optimizing the results through better understanding of the intervention–context fit ( Domecq *et al.* , 2014 ; Glasgow, 2008 ; Lyon *et al.* , 2011 ; Trompette *et al.* , 2014 ). Normative arguments concern the intrinsic value of stakeholder engagement in terms of accountability to funders and conducting research to serve public interests. Sharing expertise is an ethical mandate for stakeholder engagement in research, particularly patient engagement, as a manifestation of the democratization of the research process ( Brett *et al.* , 2010 ; Domecq *et al.* , 2014 ). Engagement can yield mutual and continual learning, which Gluckman (2014) describes as a shift from the paternalistic “science advise” model to a more democratic model. Finally, political arguments are used to justify engagement on political grounds, where engaging non-researcher stakeholders can make them feel empowered and included, thus increasing a sense of ownership. Managerial arguments refer to managers who recognize and advocate for the importance of engagement to ascertain organizational sustainability of the engagement ( Bombard *et al.* , 2018 ).

Despite the fact that research has described many advantages of stakeholder engagement and co-design, studies have also highlighted the disadvantages that can exist throughout the research process ( Fransman, 2018 ; Oliver *et al.* , 2019 ). These disadvantages concern practical costs, personal costs to researchers, for example, increased interpersonal conflicts, and professional costs such as reputational damage ( Oliver *et al.* , 2019 ). Studies have shown that co-designing can be time consuming because the process tends to take longer than expected ( Concannon *et al.* , 2012 ; Kok *et al.* , 2016 ). As an example, health professionals are often busy treating and caring for patients and have few opportunities to actively participate in the development and implementation of a research intervention ( Ben-Tovim *et al.* , 2008 ). There may also be costs to the research profession. According to Oliver *et al.* (2019 , p. 6.) “doing co-design recklessly, discourteously or without due attention to professional etiquette can cause significant ill-feeling about participating in research”. Another disadvantage is if the co-design process is based on a false appearance of engagement [tokenistic] ( Osborne *et al.* , 2016 ), yielding frustration among stakeholders, such as patients, with the consequence that they do not want to be engaged in co-design processes again ( Domecq *et al.* , 2014 ).

The engagement movement has gained increasing interest both politically, in private companies and in public institutions, for example, within the health care system, with descriptions of the concept as a “magic concept” ( Voorberg *et al.* , 2015 ). The previous section shows that it is not only a concept that can solve problems but also has the possibly to create others. In summary, the overall rationale for making use of engagement methods such as co-design is the ability of stakeholders to increase the quality of a product or an intervention, as in this project. Another rationale is efficiency, with the assumption that by engaging stakeholders in the workflow solution, parts of the task can either be streamlined or completely solved by the engaged stakeholders and thereby the workflow can be streamlined, for example, in the health care system. A third rationale is ownership with the desire for increased support and loyalty from, for example, patients and relatives, by involving them in the decisions. Engagement and co-design are rooted in social movements such as customizations and patient involvement, which is relevant to keep in mind when exploring and discussing the key challenges associated with having stakeholders take part in the WALK-Copenhagen project (WALK-Cph), which has its roots in biomedical research.

## 3. Methods

### 3.1 The WALK-Copenhagen project

The WALK-Cph ( Kirk *et al.* , 2018 ) was initiated by the research team behind this study to accomplish a multi-facetted intervention to increase mobility in older medical patients admitted to two hospitals in Denmark. The intervention was co-designed by the research team and health professionals, patients and relatives in a series of workshops ( Table 1 ) ( Sanders and Stappers, 2008 ). Previous research by this team has shown that older medical patients spend a median of 22 h per day being inactive during hospitalization ( Pedersen *et al.* , 2013 ), which is associated with increased risk of functional decline, loss of independence and death ( Brown *et al.* , 2004 ; Zisberg *et al.* , 2011 ). The intervention was thus planned to increase the mobility of patients. The WALK-Cph project was initially designed purely as a biomedical research project. It focuses on preventing diseases and death in people through physical activity ( de Vries *et al.* , 2012 ). By incorporating a co-design process and engaging health professionals, patients and relatives, the project is also designed humanistically. It is therefore an unusual case because it is uncommon that biomedical research is combined with humanistic research using iterative co-design workshops. Most researchers in the research team behind the project are used to working with type I evidence (i.e. studies that link physical activity to risk factors or health outcomes) and type II evidence (i.e. studies that link interventions to physical activity behaviour and are tested in randomized controlled trials) ( Rütten *et al.* , 2016 ). What makes the case even more unusual is that the target group for the co-design process was older patients with medical illness in need of medication and hospital care and thus potentially difficult to engage in a co-design process.

### 3.2 Study design and setting

The study used a qualitative design to explore the co-design process of the WALK-Cph intervention intended to increase mobility in older medical patients admitted to two medical departments at two university hospital in Denmark. Qualitative methods are particularly well suited for exploring and understanding co-design processes, which, for example, implies substantial attention to social interaction between stakeholders and the context ( Sanders and Stappers, 2008 ).

The WALK-Cph project was carried out in Denmark, which has a tax-funded health care system. Free treatment is provided for all citizens for primary medical care, hospital care and home-based care services. The WALK-Cph project involved four medical departments at three public hospitals in the capital region of Copenhagen, Denmark. The four departments encompass six medical specialities: (1) endocrinology; (2) infectious diseases; (3) pulmonary diseases; (4) emergency medicine; (5) gastroenterology and (6) general medicine. In total, two of the departments, endocrinology (Hospital X) and general medicine (Hospital Y), were randomized to the mobility intervention before the co-design process. The two intervention departments are situated in different hospitals (X and Y) and municipalities (X and Y) in Denmark.

The two departments were almost similar in size and staff composition. The Department of Endocrinology (Department X) has 24 beds and 36 staff members consisting of nurses ( *n* = 18), certified nursing assistants ( *n* = 6) and physicians with responsibility in the department ( *n* = 12). The Department of General Medicine (Department Y) has 25 beds and 37 staff members consisting of nurses ( *n* = 18), certified nursing assistants ( *n* = 3) and physicians with responsibility in the department ( *n* = 8). The therapists are organized differently and have different links with the departments. In Hospital X, the therapists are organized centrally in a department of occupational and physical therapy. and Department X call for therapists if a patient is in need of therapy. In Hospital Y, the therapists are staff members in Department Y.

### 3.3 Recruitment of co-design participants

The participating health professionals were recruited from the two intervention departments (X and Y) as well as from the department of occupational and physical therapy (Hospital X), the rehabilitation departments in Municipalities X and Y and the home care services in Municipality X. The reason for involving participants outside the two intervention departments was that patients are admitted for a short time, so an intervention that focuses only on hospitalization may not have much effect. Therefore, it was decided that the municipalities and the home care services should be involved. The head managers (e.g. head nurses and chief physicians) were approached to arrange access to staff, patients and relatives in the departments. The frontline managers ( Hasson *et al.* , 2014 ) involved in the design process were charge nurses, ward physicians and charge physiotherapists from the departments. The frontline managers identified nurses, nursing assistants, therapists and physicians who had experience with or would be interested in participating in designing and implementing an intervention that could accommodate the clinical outcome of the intervention (an increase in upright time by 45 min a day). In total, one physician, three nurses, three nursing assistants, three physiotherapists, three occupational therapists and five frontline managers participated in the co-design process ( Table 2 ).

We included older medical patients (+65 years) hospitalized via the emergency departments at the two hospitals. We excluded patients who were not able to walk, in isolation, not able to collaborate (e.g. due to dementia), not able to understand and speak Danish, undergoing cancer treatment or terminally ill. We included relatives who had a relative, friend or family member (+65 years) admitted via one of the two emergency departments and transferred to one of the intervention departments. When a patient consented to participate in the project, he/she was asked if he/she and his/her relatives wanted to attend a workshop where they would be given the opportunity to report on their experience regarding mobility during hospitalization and after discharge, as well as make suggestions about what actions could help them become more mobile during hospitalization. In this recruitment process, the patients and their relatives were made aware of their role, how they could contribute and the costs in terms of time ( Jolibert and Wesselink, 2012 ).

In total, 13 patients were invited, and seven patients accepted to participate in the workshop ( Table 1 ). In total, two of the patients had relatives who agreed to participate in the same workshop. To secure maximum variation in the representation of both relatives and patients, we contacted a patient council in the Capital Region of Denmark and asked if any of them could participate in the workshop. In total, three persons responded positively, and all complied with the inclusion criteria of having relatives (+65 years) who had recently been hospitalized via the emergency department and transferred to a medical department. On the day of the workshop, one patient cancelled due to readmittance to the hospital, and one relative participated without her older relative, who had died. In total, five patients and five relatives participated in the workshop ( Tables 1 and 2 ).

A total of five members from the research team participated in all workshops (JWK, MMP, TQB, RB, OA). Of the 11 members of the research team, seven were health professionals (nurses, physiotherapists and physicians) with 1–40 years of experience working within the health care system. The team also included a statistician, an expert in implementation science and an expert in anthropology. MMP and JWK were formal project managers of the WALK-Cph project. The research team consisted of one research assistant, two PhD students, three postdocs, one associate professor and four professors. JWK, who had overall responsibility for leading and facilitating the workshops, is a health professional (nurse) with experience as a process consultant. In the next section, workshops are described as a contextual framework for follow-up discussions in the research team both after the workshops and at weekly Friday meetings throughout the co-design process.

### 3.4 Workshops

The stakeholders worked together in five workshops that took place between March 2017 and September 2018 ( Table 1 ). The workshops were interactive ( Pavelin *et al.* , 2014 ) with the aim of encouraging creativity and producing ideas for the proposed intervention. Interactive workshops are defined as “a structured set of facilitated activities for groups of participants who work together to explore a problem and its solutions, over a specific period of time, in one location” ( Pavelin *et al.* , 2014 ).

Before the workshops, the research team developed semi-structured guides to support the team members. The researchers acted as facilitators of smaller groups during the workshops to ensure that the same topics were discussed in all groups. The topics were inspired by evidence-based literature on mobility ( Brown and Flood, 2013 ) and our findings from the initial baseline and observational studies ( Kirk *et al.* , 2019 ).

The five workshops each lasted 3–4 h and were held in a meeting room at Hospital X. The aim of the workshops was to conduct co-design sessions in which the stakeholders could contribute to designing the intervention facilitated by the research team who coordinated and assisted group discussions and activities during the workshops ( Sanders and Stappers, 2008 ). The facilitators did not contribute with ideas but encouraged input from participants in their respective groups. The research team also had the task of performing feedback loops by presenting data from the baseline studies (accelerometer data and observational data). These were used as mirror data in the workshops and were defined as data representing current practice ( Kerosuo *et al.* , 2010 ).

The participants in the workshops were divided into smaller groups depending on the topics discussed. In workshop 2, for example, the patients and their relatives were separated when discussing which activities could have increased the possibility that they would get out of bed and walk during hospitalization. We wanted to ensure that the relatives did not influence the patients and that the patients' voices were heard. Our initial observations showed that some relatives encouraged patients to stay in bed because they thought the patients were too ill to walk, even though the patients themselves wanted to get out of bed and walk.

Flip charts, PowerPoint presentations and sticky notes were used during the workshops and were considered mediating artefacts for the social interaction and part of the context in the workshops ( Pavelin *et al.* , 2014 ). Flip charts were used to note all suggestions on how the intervention could be designed to ensure that patients would walk during hospitalization and after discharge. All discussions and interactions in the five workshops were video- and audiotaped.

In between the workshops, MMP and JWK were in frequent contact with the frontline managers from intervention departments X and Y, via telephone, emails and meetings, to secure management support. This encompassed discussions on which physical changes in the department would be necessary to accommodate a given intervention component (e.g. where and how a walking path could be placed most appropriately). In this process, it was decided that the final workshops should be held separately for the two departments, primarily to ensure micro-level adaptation and second because department Y was due to go on strike in connection with a national labour conflict being negotiated at that time. The micro-level adaptations led to minor department-specific adjustments to comply with different regulations in the two hospitals.

### 3.5 Data collection and analysis

The analysis is based on data collected mostly during follow-up discussions and at weekly Friday meetings of the research team and on part of the transcribed data from the workshops. In total, 64 A4 pages were used in the analysis.

The analysis was based on repeated readings of the transcribed material by JWK ( Bundgaard *et al.* , 2018 ). Thematic content analysis ( Graneheim and Lundman, 2004 ) was conducted with a background in the analytical question: What key challenges arise in the material in relation to the co-design process? The process of analysis included condensing codes and categories according to meaning and finally themes were identified in the material (an example is provided in Table 3 ). The themes were subsequently discussed with PN and then with the other research team members before consensus was reached ( Table 4 ). An issue was determined to be a theme if it fulfilled three criteria: (1) a considerable amount of time and attention was devoted to the issue in the workshops, as documented in written notes and/or oral discussions; (2) the issue was considered important in workshop discussions for designing an effective intervention and (3) the issue was perceived by the research team as a challenge that was not easy to resolve, e.g. the right way to include older medical patients and relatives.

### 3.6 Ethical considerations

The Danish Data Protection Agency (AHH-2016-080, I-Suite no. 05078) approved the study, which was funded by the Velux Foundations (F-21835-01-04-03), the Association of Danish Physiotherapists (PD-2018-30-10) and the Capital Region of Denmark (P-2018-2-11). The project adheres to the directives of the Declaration of Helsinki ( The Nuremberg Code, 1949 ). Anonymity was achieved by assigning stakeholders a code instead of using their full names in the field notes. The researchers maintained a confidential file of identifiers tied to the stakeholders' backgrounds, so that the workshop data (recordings and transcripts) could be coded as a basis for in-depth analysis. Before participating in the workshops, all stakeholders were informed about the aim of the study and were assured that participation was voluntary and that they and the results would be pseudo-anonymized. Because the head and frontline managers had approved the health professionals' participation in the workshop, written informed consent was not obtained from them; written informed consent was obtained from the patients and relatives. All stakeholders were given the opportunity to withdraw from being followed in their daily work, but none of the stakeholders did so.

## 4. Results

In this section, we present the results from the point of view of the research team, who were tasked with engaging and running the co-design process. In total, two themes emerged in the analysis: engagement and facilitation. Engagement refers to different challenges in recruiting stakeholders in the co-design process. Facilitation refers to different challenges for the research team with regard to changes in roles and activities. The theme engagement consists of two sub-themes: recruiting patients and involving physicians. Facilitation consists of three sub-themes: adjusting to a new researcher role; utilizing contextual knowledge and handling ethical dilemmas ( Table 4 ).

### 4.1 Engagement

The challenge of engagement in the co-design process concerned recruiting patients and involving physicians.

#### 4.1.1 Recruiting patients

An important challenge was to identify suitable patients and relatives and recruit them for participation in the co-design process. Many practical questions arose in this phase; for example, “How do we get the ‘right’ patients to participate?”, “What do they need to know?”. There were also questions of an ethical nature, including “What degree of engagement do they want?”, “How can we ascertain collaboration and maintain respect for each other’s viewpoints?”, “What is legitimate to talk about?” and “Who controls this process of co-design?”.

About half of the patients who were approached declined to participate in the workshop. Reasons for declining included having a low level of functional capacity and/or experiencing aggravation of their disease, and some had been readmitted. Some patients were concerned about how to transport themselves to the workshops. To overcome this inclusion barrier related to practicalities, we offered all patients and relatives the option to be picked up by taxi or a research staff member (in a hospital car) and brought home after the workshop. We also prepared written materials about mobility and what was going to happen during the workshop in layman's language. A total of four days before the workshop took place, reminders were sent to patients and relatives by e-mail or telephone about the agreement to participate. Some relatives agreed to participate because they had negative experiences with the health services, and they felt there was a lack of focus on getting older patients out of bed. A relative expressed

My mom was only out of her bed one time when hospitalized for three days. This was even though she could walk well. I do not think the staff focused on how important activity is for older people. I would like to participate and contribute with solutions to handle the problem (relative, department X).

Being invited to a workshop focusing on activity for older patients made them appreciate the opportunity to be involved in solving these problems.

#### 4.1.2 Involving physicians

The other stakeholders considered it was fundamental that physicians were responsible for delivery of some intervention components to achieve the desired clinical outcome. The physicians' response when presented with the project was generally positive. Even though we tried to recruit at least two physicians from both departments, only one physician participated in the workshops. Further, the physician only participated part of the time in two of the three workshops due to clinical work.

We approached the physicians via numerous emails and telephone calls, but they did not respond. We also asked the frontline managers if they could help us identify physicians for potential inclusion in the workshops. When asking the participating physician if she had any idea why her physician colleagues did not want to participate, she replied

Many do not think they have time to participate in workshops. Not because they do not think activity is an important topic, but many also think it is the physiotherapists' responsibility to get patients out of bed (physician, department X).

With a background in these experiences, we decided to carry out interviews focusing on the barriers and facilitators experienced by physicians because we considered it important for continuation of the project ( Pedersen *et al.* , 2020 ). This was not part of the initial design of the project. It took time and resources, causing delays to the project.

### 4.2 Facilitation

The challenge of facilitation in the co-design process involved adjusting to a new researcher role, utilizing contextual knowledge and handling ethical dilemmas.

#### 4.2.1 Adjusting to a new researcher role

The co-design process was intended to give the participants a strong voice, with the researchers facilitating the participants rather than acting as experts. This meant that we as researchers had to give up a great deal of the control we are used to when conducting research involving health professionals and to accept the stakeholders' experience-based knowledge as just as important and relevant as knowledge based on research *.* A recurring question raised in the research team was “what do we do if it turns out that the intervention components that are proposed and selected are not based on evidence? How can we be sure it works?”. The researcher role was not only at odds with our traditional role as experts who rely on generalizable research-based knowledge when designing, implementing and evaluating interventions but thus also at odds with what researchers normally think counts as relevant knowledge. The new role led to much debate among the researchers concerning our new position in relation to the stakeholders, the transfer of power from us to them and how we should act and interact with the stakeholders. These discussions led to didactic decisions about introducing ourselves by name and professional education, for example, physiotherapist and nurse, and not by our academic positions (e.g. postdoc or professor) to facilitate dialogue on more equal terms with the participants.

#### 4.2.2 Utilizing contextual knowledge

The importance of contextual knowledge became obvious during and after finishing the co-design process in the two intervention departments. In total, four of the researchers (MMP, JWK, OA, TQB) had many years of experience at Hospital X where one intervention department was located and thus were familiar with the physical and organizational context and had experience concerning how to act and interact in this organizational culture. Furthermore, the physical proximity of this department made it possible to quickly pay a visit if circumstances had changed and/or something needed to be addressed. Hence, drawing on extensive contextual knowledge made it easy to monitor the intervention and implementation process and maintain the relationship and trust in this department.

Such contextual knowledge in the research team was limited with regards to the intervention in Department Y, located at Hospital Y. It took a great deal of time to find out, for example, who was the right person to contact when materials had to be developed or who to talk to regarding different issues. This became evident when the co-design process entered its final phase. The research team had contacted the architect from Department X who participated in the design workshop and contributed with design suggestions such as colours and size of the walking path. It was not clear, however, who was responsible for architectural issues in Hospital Y, and it turned out to be a space manager organized in a completely different department at the hospital. Likewise, even though both departments were located in the same region, the board of directors decided that same colours could not be used in the two intervention departments. This lack of contextual knowledge appeared as a lack of established relationships between the stakeholders and the research team just as the physical distance proved to be an important challenge. This meant that project coordination was significantly more time consuming in Department Y than in the Department X, where we could utilize our contextual knowledge.

#### 4.2.3 Handling ethical dilemmas

Several ethical dilemmas emerged in the co-design process, providing considerable challenges because they needed to be addressed for the project to proceed as intended. The lack of physician engagement led to some ethical questions: “How can we ensure that we include the physicians' perspectives in the project?” and “Can we collaborate with the physicians if they do not respond?”. There were many discussions among the researchers on how to handle such ethical dilemmas. Another ethical dilemma concerned our role as researchers in the co-design process. We believed that our initial discussions about this dilemma meant that it was handled appropriately and could thereafter be put to rest. However, the issue of our role as researchers emerged on many occasions, leading to many ethical questions being discussed among the researchers: “What is our goal with the project?”, “Who should influence what and why?” and “How to facilitate in the right way so that all stakeholders were encouraged at all levels of creativity?”.

A further ethical issue concerned the balance between “process” and “product”. Our goal was a trial and the clinical outcome (i.e. the “product”), and the co-design process was for us a means of achieving this goal. Researchers in biomedical research usually control an intervention design process, but in a co-design project, this process to some extent becomes a “product” because it is an important objective for the co-design project. We struggled with not being able to fully control the co-design process, which as a design is based on somewhat uncontrollable processes. Dilemmas occurred when we as a research team wished to decide on issues, for example, adding new components to the intervention because this was inconsistent with our initial design where we as researchers should take on a facilitating role. Such decisions challenged the democratizing foundation embedded in co-design processes. Consequently, it required more work to maintain trust between the stakeholders and the research team.

Overall, the theme facilitation concerned a more general paradigmatic contrast between different research approaches: what is considered evidence? and what counts as real and relevant knowledge?. This is elaborated further in the discussion.

## 5. Discussion

This study identified two key challenges, engagement and facilitation, from the point of view of the research team when engaging in and running the co-design process of the WALK-Cph intervention with patients, relatives and health professionals. Both advantages and disadvantages of stakeholder engagement have been explored from political, health and research perspectives ( Oliver *et al.* , 2019 ; PCORI, 2010 ; Richards, 2014 ). In this project, these perspectives are operationalized by the political discourse on stakeholder engagement having an impact on fund announcements and demands from executive boards when new initiatives are to be developed. One problem may be that engagement and involvement has become “the good thing itself”, which makes it difficult to be critical as well. The positive approach can mean that the co-design values (democratization, equality, we are all experts) do not actually come into play. Although it is increasingly encouraged to engage stakeholders in co-design processes ( Armstrong *et al.* , 2018 ; Domecq *et al.* , 2014 ; Richards, 2014 ), our findings show that this may be difficult.

The disadvantages of engaging stakeholders, particularly patients, have been established in previous research ( Klesges *et al.* , 2005 ; Légaré *et al.* , 2010 ; Stewart *et al.* , 2011 ), but there is no evidence for a particular approach on how best to identify or select patients for engagement ( Domecq *et al.* , 2014 ; Mockford *et al.* , 2012 ; Nilsen *et al.* , 2006 ). We experienced difficulties in recruiting older medical patients to the co-design workshops, something which has been addressed in the literature ( Bombard *et al.* , 2018 ; Carman *et al.* , 2013 ; Domecq *et al.* , 2014 ). These problems existed despite the fact that we invited patients to take part in workshops, which allow for a high degree of engagement and interaction, instead of interviews or surveys, which are the most common methods used in health care to engage patients and relatives ( Domecq *et al.* , 2014 ). Workshops allow for a higher degree of engagement than interviews and surveys if the engagement is not handled tokenistically ( Minogue and Girdlestone, 2010 ; Osborne *et al.* , 2016 ). It may be that the workshop method requires too much time, resources and human capacity from patients and relatives, also when taking into consideration that our goal was to engage older medical patients and their relatives. A semi-structured focus interview or single interviews might have provided the opportunity to recruit more patients but at the expense of high engagement and the opportunity to hear and respond to other stakeholders' thoughts ( Carman *et al.* , 2013 ).

In our study, the physicians' lack of engagement in the co-design process was a considerable challenge. All stakeholders, including the research team, agreed that the engagement of physicians in the intervention was important because of their authority and (formal and informal) leadership roles in relation to motivating patients to get out of bed. Balint (1988) refers to the concept of “the doctor–drug” which describes how the mere presence of a physician can influence patients' responses to illness and treatment. There can be many reasons for their non-engagement, for example, the co-design idea does not make sense to physicians and they may not perceive mobility as an area of responsibility for them. We have previously found that physicians tend to focus on mobility primarily when it concerns patient flow, discharge or transfer to other departments ( Kirk *et al.* , 2019 ). However, research has shown that the physicians' advice on mobility has an important influence on increased mobility in patients ( King, 2010 ; So and Pierluissi, 2012 ). Oliver *et al.* (2019) have described “how co-production can lead to research outputs that are regarded as being of lower quality than ‘real’ or ‘pure’ research” (p. 5), potentially being seen as a threat to physicians’ research career. However, the empirical material in this study cannot support this statement. Despite the physicians' reluctance to become engaged, we did not experience that their lack of engagement hindered the workshops or the co-design process. However, their absence had the consequence that the other stakeholders (managers, nurses, nursing assistant and physiotherapists) had to discuss and select intervention components that they believed and had experience-based expectations would motivate the physicians and which the physicians should agree to be responsible for. In retrospect, the research team agreed that the high importance put on physician engagement might have been too one-sided. The low engagement from the physicians suggests that the responsibility for the proposed intervention should have been allocated more evenly among all the health care staff ( Hall, 2005 ). The randomization of the departments before initiating the co-design process can be viewed as a disadvantage in relation to engagement and participation in the co-design process. It might be that the departments that were randomized to control departments had previous experience with co-design processes and therefore could have provided physicians with co-design experience and engagement. On the other hand, randomization before initiation of the co-design process ensured that all stakeholders participated in the design of an intervention that they were to carry out themselves and in which they would therefore (theoretically) engage. In general, engagement challenges are well known in the literature, but discussing this issue took up considerable time and required a great deal of mental capacity at the Friday and follow-up meetings.

The other key challenge, facilitation, is recognized as a special area of expertise ( Pirinen, 2016 ). Facilitation can be achieved using techniques such as leading, guiding and scaffolding ( Sanders and Stappers, 2008 ). The facilitator also needs to take flexible roles during the process, alternating between patient orientation, solution orientation and systems thinking ( Pirinen, 2016 ). Tollyfield (2014) describes how facilitation in co-design projects catalyses receptive contexts that encourage engagement by creating a positive environment with mutual respect and equal partnership. In the co-design process, mutual respect and equal partnership means that stakeholders share power with the researchers and have considerable responsibility for the design of the intervention. Kramer *et al.* (2010) stress the importance of honouring, trusting and respecting the stakeholders' knowledge and expertise and taking their needs and priorities into account. Stakeholders' influence is considered crucial for successful implementation of interventions due to greater ownership ( Boaz *et al.* , 2018 ). The question is whether or not honouring, trusting and respecting the stakeholders' knowledge and expertise conflict with the idea of equal partnership (and democratization) between stakeholders and researchers. Accounting for the stakeholders' experience-based knowledge can challenge the researchers' perspective on knowledge. This was perhaps most notable among some of the researchers in our study, who primarily had experience with type I and II evidence related to a nomothetic understanding of science, where objectivity and acontextuality are key factors ( Møhl and la Cour, 2008 ) and for whom power of evidence-based knowledge was hard to surrender. Most of the researchers in the study were used to conducting randomized controlled trials, performing statistical analyses and using quantitative methods and protocols, which are usually carefully planned and defined before the start of a project ( Kramer *et al.* , 2010 ). However, working in co-creation processes requires acceptance of knowledge that is relational, contextual, experience-based and situational, that is, more of an idiographic understanding of science ( Sjørslev, 2015 ). This focus on evidence-based knowledge is not only a characteristic of the researchers in this study but a more fundamental problem in knowledge formation. In present-day western society, “objective” evidence and numbers are generally considered “reality” to a greater extent than countless experience-based knowledge ( Hastrup, 2004 ). As Graffy (1999) has described, these different perspectives require personal qualities and skills that often need to be learned, including among researchers. We learned that researchers need to discuss different forms of knowledge, the degree of engagement of stakeholders and outcomes before embarking on co-design processes.

The different perspectives on knowledge also relate to differences in power structures and status within health care where professionals occupy more dominant positions than members of the public and patients ( Martin, 2008 ; Ocloo and Matthews, 2016 ). Working with co-design processes requires that power is transformed from what Pitkin (1973) called “power over” to “power to”. The former is having power over another person, whereas power to denotes capacity, potential, ability or wherewithal ( Pitkin, 1973 ). Traditionally, in biomedical research, researchers set the agenda and design the interventions, and stakeholders, for example, health professionals, are responsible for implementing them ( Domecq *et al.* , 2014 ). In researcher-led processes, stakeholders have limited influence because protocols and rules are determined by the researchers ( Jolibert and Wesselink, 2012 ). Our initial decision to facilitate with a hands-off approach in the design of the intervention and give power to the stakeholders provided a challenge because it was difficult for the research team to let go of control. Kramer *et al.* (2010) have emphasized the importance of allowing the design of the protocol to be flexible and have pointed to the relevance of having discussions on power before starting a co-design project. We chose the workshop format to give the participants more influence, knowing that it would challenge our familiar roles as researchers. We could have chosen the laboratory format, which is characterized by controlled conditions and is well known within biomedical research. This format would have maintained the research team in their role and not given the stakeholders the opportunity to work creatively, exploratively and with such a high degree of focus on the intervention.

The ability to systematically utilize knowledge about the local context proved to be another challenge. In general, this type of knowledge is important when implementing interventions ( Davidoff, 2019 ; Doran *et al.* , 2012 ; McCormack *et al.* , 2002 ; Tomoaia-Cotisel *et al.* , 2013 ). Associated with contextual knowledge is the building of trust with stakeholders, which is important to ascertain a well-functioning collaboration between the research team and the stakeholders throughout the project. Hastrup (2004) points out that to establish a true relationship, the parties must be present in the same space; this has to be theirs (the stakeholders) if relational trust has to be created. We strove to build trust through ongoing interactions and individualized communication with stakeholders to address their concerns ( Hinchcliff *et al.* , 2014 ; Mallery *et al.* , 2009 ). We learned about the importance of setting aside time at the beginning of projects to become familiar with the local context. Building trust was more easily achieved in one of the intervention departments due to our contextual knowledge gained through previous research carried out in this environment ( Carman *et al.* , 2013 ).

Ethical challenges also emerged in the co-design process, including acknowledging the physicians' perspective in the project even if they did not respond, balancing between process and product, balancing between a hands-off and a facilitating role and balancing between stakeholders' experience-based knowledge and the researchers' urge to refer to evidence. Simonsen and Robertson (2012) have described how ethical issues always have to be addressed in designs that involve stakeholders, and they provide examples of ethical questions that can guide ongoing reflection and iteration during a design process, for example, “Do users actually have decision power? If so what kind?” or is it a kind of "pretence" that we are equal and our knowledge has equal worth? Our findings suggest that both the research team and the physicians operated in “an older paradigm of a paternalistic clinician system” ( Simonsen and Robertson, 2012 , p. 228), where the experts know best.

In this project, the aim of the “making” part was an intervention and not a commercial product. One argument supports a difference between what Filipe *et al.* (2017) call the right-based argument. This holds that people, the patients in this project, have a right to participate in decisions that directly affect them, for example, when they are admitted to a hospital. As a citizen, you can choose not to buy a commercial product. According to Filipe *et al.* (2017) , there is a quality argument in which the patient and relatives' experiences with illness, health services and treatment contribute to improvement, knowledge and health research. Therefore, designing the intervention in a co-design process can be considered a means to achieve broader human goals and tackle complex societal problems and therefore is in the public interest. Also, a main point is that the difference between a commercial product and a public product (health intervention) is that there is no articulated profit motive with the intervention in the study. But the question is whether savings and rationalization are at stake beneath the surface, and through that, economic logics are also sneaking into health care interventions. Both companies and health care have been slow to adopt co-design, using similar arguments in terms of these processes being threats to the hierarchies of existing professionals. Whereas companies consider co-design as an academic endeavour with little business relevance ( Sanders and Stappers, 2008 ), health care researchers may want to avoid novel approaches and have prejudices towards unconventional methods ( Bason, 2014 ).

This study has shortcomings that need to be considered when interpreting the findings. One limitation was the small population of patients and relatives participating in the workshops, which likely affected the co-design process. If we had engaged more older patients and their relatives, we could have organized one more workshop to give the patients a stronger voice in the project. Instead, we took the decision later in the project to interview 20 patients who tried out the intervention to obtain their perspective on what worked, why and for whom ( Stefánsdóttir *et al.* , 2021 ). Another limitation is that we did not systematically examine what factors the patients perceived to be most effective in recruiting them to participate in co-design processes. We could probably have engaged more patients if we had gone to voluntary organizations in Denmark (e.g. Ældre Sagen [DaneAge Association]), but we decided against this because those patients would not be truly representative of the target population, and they might have had personal agendas ( Domecq *et al.* , 2014 ). Another limitation is that the patients who participated in the co-design process have not been able to test the intervention. It took considerable time to develop the intervention to the testing stage, so it was not possible for the research team to maintain contact with all the participating patients and relatives. A further limitation is that we describe challenges from the research teams' perspectives, which means that the challenges may have been viewed differently by other actors.

In conclusion, we identified two key challenges associated with having health professionals, patients and relatives co-designing an intervention to increase mobility in older medical patients admitted to a hospital in Denmark. The challenges were related to engagement and facilitation. Despite these challenges, we believe that designing interventions in co-design processes is an important part of biomedical research that aims to ultimately influence clinical practice. Biomedical research will need to vie with humanities research, which is a challenge discussed throughout the article.

Based on our findings, we recommend that it is not only patients and relatives who need to be prepared to be part of stakeholder engagement and design processes. Researchers who want to use co-design must be prepared for the extra time required and the need for “engagement literacy”, that is, skills concerning communication, facilitation, negotiating and resolving conflict. We also recommend that research managers in biomedicine recognize both the importance of relational knowledge, which a co-design project can contribute, and their management support in the form of facilitating conflicts as well as fact that the project may take longer. We agree with Reevers (2010) who stated that collaboration “is not a gift from the gods but a skill that requires effort and practice” also for researchers. Time is also required for ethical discussions and considerations concerning knowledge creation.

## Figures and Tables

**Table 1:** Workshops

Workshop	Date	Participants	Focus/Aim/Contents	Comments
I	5 March 2017	Health professionals	Discussions on potential intervention components. The participating health professionals suggested 15 intervention components	15 intervention components were considered too comprehensive. Therefore, a modified Delphi method was undertaken by the research team
II	20 September 2017	Patients and relatives	Discussions on potential intervention components. Patients and relatives were presented with the preliminary intervention developed in workshop I, and some of the components were excluded. The participants suggested 28 components for the intervention, including components suggested by health professionals from workshop I	A Delphi method similar to the one held after workshop I was performed by the research team. Based on this, ten interventions components were identified
III	5 December 2017	Health professionals and managers (same as in workshop 1)	The preliminary ten-component intervention was presented. Also, the process of inclusion and exclusion of components was presented along with quotes from patients and relatives to ensure that the patients' voices were heard and to acknowledge patients, relatives and health professionals as experts in their own lives and work situations	Finally, consensus was reached about seven components: a walk path; the physicians prescribe walk plans; independent collection of clothes and beverages; posters and a welcome folder encouraging patients to walk and exercise; after discharge, patients with a walking plan, who are discharged with a rehabilitation plan, will be contacted by phone by a municipal therapist; after discharge, patients with a walking plan, who are discharged without a rehabilitation plan but receive home care, will be contacted by phone by home health care personnel
IV	31 May 2018	Health professionals, the research team and a graphic designer	During the workshop, the health professionals and managers were asked to discuss barriers and facilitators for all ten components until consensus was reached. The final intervention consisted of seven components to be tested in the departments	The drafts for the walking path, the walking plans and the posters were sent to the patients and relatives who participated in workshop II to give them an opportunity to comment on the design of these components. Four of ten responded with the consequence that the red colour on one of the walking plans was darkened, and the text on the posters was enlarged to make it more readable for older patients
V	5 September 2018	Health professionals, the research team and a graphic designer (same as in workshop IV)	?	The final intervention consisted of seven components to be tested in the departments

**Note(s)** : The graphic designer's role was to collaborate with the participants on the design of all objects that were part of the intervention, e.g. ensuring that the colours of the walking path and the chairs adhered to regulations, ensuring that the chairs were of the correct size, helping in the development of posters and walking plans and providing graphic inspiration on the design of the walking path. The municipality was not able to participate in the workshops because they had to prioritize their resources for the implementation of a new information technology (IT) system. Therefore, MMP and JWK met with the participants from the municipality to discuss decisions from workshop IV and agreement was reached on the components involving the municipality

**Table 2:** Participating health professionals

Profession	Number	Years of experience	Gender
Physiotherapists	3	<2, >5, >5 and >5	4 females
Physician	1	>10	1 female
Occupational therapists	3	>5, <2 and >2	3 females
Nurses	3	<5, >5 and >10	3 females
Assistance nurses	3	>5,>10 and >10	3 females
Frontline managers	4	>5, >10, >10 and >10	1 male and 3 females
Patients	5		3 male and 2 females
Relatives	5		1 male and 4 females

**Table 3:** Examples of the abstraction process of the co-design process in the WALK-Cph intervention development

Meaning unit	Condensed meaning unit. Description close to the text	Condensed meaning unit Interpretation of the underlying meaning	Sub-theme	Theme
One colleague asked how we secured to get the right patient to participate in the workshop	Getting the right patient to the workshop	In connection with recruitment, a concern is raised that the patients do not represent the group we want, namely the vulnerable patients with multi-morbidities	Recruitment of vulnerable patients	
A colleague has experienced that the patient was concerned about how to transport herself to the workshops as she could not drive her car at the moment because of her illness	How do the patient transport herself to the workshop	When the target group for workshops is frail patient, we are responsible, as part of the recruitment process, to include help with transport to the patients	Recruiting vulnerable patients	*Engagement*
Others have experienced that the patient was unsure if he had anything to contribute with due to his illness when the topic for the workshops was physical activity	Concerns about having anything to contribute with in the workshop	The patient's current disease situation meant that the topic of physical activity was perceived as something abstract and which was difficult to contribute to	Uncertainty about contribution	

**Table 4:** Final sub-themes and themes

Sub-theme	Theme
Recruiting vulnerable patients	*Engagement*
Involving physicians	
Adjusting to a new researcher role	*Facilitating*
Utilizing contextual knowledge	
Handling ethical dilemmas	
